# Difficulties in emotion regulation mediate the association between sensory over-responsiveness and motor coordination symptoms in community-dwelling midlife adults: a cross-sectional analysis

**DOI:** 10.3389/fpsyg.2026.1779854

**Published:** 2026-05-29

**Authors:** Merav Asher, Hagit Hochner, Tamar Bar-Shalita, Maayan Agmon

**Affiliations:** 1 School of Nursing, Faculty of Health and Social Welfare, University of Haifa, Haifa, Israel; 2 Braun School of Public Health, Hebrew University of Jerusalem, Jerusalem, Israel; 3 Department of Occupational Therapy, Gray Faculty of Medical and Health Sciences, Tel Aviv, Israel

**Keywords:** community-based sample, developmental coordination disorder symptomatology, difficulties in emotion regulation, midlife adults, sensory over-responsiveness

## Abstract

**Introduction:**

Developmental Coordination Disorder (DCD) is a neurodevelopmental condition characterized by motor coordination difficulties that impair daily functioning across the lifespan. Sensory over-responsiveness (SOR) frequently co-occurs with DCD; both conditions are associated with elevated anxiety and depression. However, the interplay among SOR, motor coordination difficulties, and emotional functioning remains under examined in midlife adults, a period marked by neurobiological and psychosocial transitions that may place greater demands on regulatory and motor systems. Moreover, no study has investigated difficulties in emotion regulation, as distinct from emotional symptoms, as a potential mechanism linking SOR and DCD symptomatology, particularly in community-dwelling adults whose motor coordination difficulties may not reach diagnostic thresholds. This cross-sectional study examined whether SOR is associated with DCD symptomatology in community-dwelling midlife adults, and whether this association is mediated by difficulties in emotion regulation.

**Methods:**

Eighty-nine participants from the Jerusalem Perinatal Study birth cohort (mean age = 45.59 years, SD = 0.68, range 44.33–46.87; 47% women) completed the Sensory Responsiveness Questionnaire (SRQ), the Adult Developmental Coordination Disorders/Dyspraxia Checklist (ADC subscales B and C), and the Difficulties in Emotion Regulation Scale (DERS). Mediation analysis (PROCESS Model 4; 5,000 bootstrap resamples), controlling for physical activity.

**Results:**

SOR significantly predicted DCD symptomatology (total effect: B = 0.343, SE = 0.105, *p =* 0.002). When difficulties in emotion regulation were included as a mediator, the direct effect was no longer significant (B = 0.151, SE = 0.095, *p =* 0.114), while the indirect effect was significant (B = 0.192, 95% CI [0.084, 0.323]), consistent with full mediation.

**Discussion:**

These findings provide initial evidence that difficulties in emotion regulation may constitute one pathway linking SOR and DCD symptomatology in midlife, a previously unexamined mechanistic association with implications for age-appropriate assessment and intervention. Longitudinal research is needed to establish directionality.

## 1 Introduction

Developmental Coordination Disorder (DCD), also known as dyspraxia, is a neurodevelopmental condition defined by four DSM-5 criteria: (A) acquisition and execution of coordinated motor skills is substantially below expected levels given chronological age and opportunity for skill learning; (B) motor skill deficits significantly interfere with activities of daily living and impact academic or occupational achievement; (C) onset of symptoms occurs during the early developmental period; and (D) motor skill deficits are not better explained by intellectual disability, visual impairment, or neurological conditions affecting movement (American Psychiatric Association, 2013). DCD affects approximately 5–6% of school-age children globally and persists in adulthood in 50–70% of cases, rendering it a significant lifelong condition (Blank et al., 2019; Tal Saban and Kirby, 2018). As a neurodevelopmental condition, DCD is typically managed through occupational therapy, task-specific training, and functional interventions (Blank et al., 2019; Kirby et al., 2008).

Motor difficulties in DCD manifest across a range of daily activities, including driving, handwriting, balance maintenance, and fine motor tasks (Blank et al., 2019; Kirby et al., 2013). These difficulties are not remediated through maturation alone: adults with DCD exhibit significantly greater variability in walking patterns than neurotypical adults (Du et al., 2015) and particularly struggle with acquiring new skills that demand complex or automatic processing, such as learning to drive (Kirby et al., 2011), resulting in compromised occupational functioning (Kirby et al., 2013).

The Environmental Stress Hypothesis (ESH; Cairney et al., 2013), provides a theoretically grounded account of the psychosocial consequences of persistent motor difficulties: repeated motor failures, exclusion from physical activities, and chronically diminished self-efficacy generate cumulative stress that gradually erodes psychosocial functioning. Consistent with this framework, adults with DCD demonstrate significantly elevated anxiety and depression compared to controls, effects that remain robust after controlling for physical activity levels (Hill and Brown, 2013), as well as executive function difficulties and reduced self-efficacy (Blank et al., 2019; Green et al., 2006). Erskine et al. (2024) recent integrative review of 38 studies supported the ESH across the full motor skill spectrum and identified multiple mediating pathways, including interpersonal conflicts (e.g., peer problems) and intrapersonal struggles (e.g., sensory processing difficulties, executive function deficits). Of these, only five empirical studies focused on adult populations, highlighting a significant gap in literature (Erskine et al., 2024).

While childhood factors underlying DCD have been the focus of considerable research (Blank et al., 2019; Wilson et al., 2013), considerably less is known about how these difficulties present across adulthood, and the midlife period remains virtually unexamined (Meachon et al., 2022; Tal Saban and Kirby, 2018). This gap is significant because midlife is characterized by normative neuromotor changes that may interact with pre-existing motor coordination difficulties. For example, processing speed declines significantly from midlife onwards (Salthouse, 2009), and progressive volume loss occurs in the prefrontal cortex and cerebellum, structures central to both motor planning and executive control, accompanied by functional and behavioral changes (Seidler et al., 2010). Seidler et al. (2010) further propose that ageing adults increasingly recruit prefrontal resources to compensate for declining motor automaticity, placing heightened demand on prefrontal systems that are themselves undergoing age-related volumetric decline. Adults with persistent motor coordination difficulties, who already contend with executive function deficits (Blank et al., 2019; Tal-Saban et al., 2012), may be particularly susceptible to this supply-demand imbalance during midlife. This imbalance may accelerate functional decline in domains requiring integrated sensorimotor and cognitive control (Seidler et al., 2010), a possibility that has not yet been empirically examined.

These midlife challenges, moreover, may not be limited to adults with a formal DCD diagnosis. Contemporary frameworks increasingly conceptualize neurodevelopmental conditions as existing along continuous dimensions rather than as discrete diagnostic categories (Astle et al., 2022). From this perspective, motor coordination difficulties associated with DCD vary in severity across the population, and sub-clinical presentations may nonetheless carry functional significance (Blank et al., 2019; Hill et al., 2016; Lingam et al., 2010). The dimensional approach motivates the present study’s use of the term *DCD symptomatology* to denote self-reported motor coordination difficulties assessed continuously, rather than categorical diagnostic status. Participants in community-based samples are not clinically diagnosed; accordingly, the present study examines self-reported DCD symptomatology rather than confirmed DCD status. This approach supports investigation into samples where the full range of coordination ability can be examined (Hill et al., 2016; Lingam et al., 2010).

Within this dimensional framework, one co-occurring factor of relevance to DCD symptomatology is Sensory over-responsiveness (SOR), characterized by exaggerated responses to non-painful stimuli resulting in heightened arousal and avoidance behavior (Bar-Shalita et al., 2019; Miller et al., 2007). Research in children has established that sensory processing difficulties frequently accompany DCD, with SOR emerging as particularly prominent (Allen and Casey, 2017; Mikami et al., 2021). The relevance of sensory processing to motor coordination is supported by converging evidence: proprioceptive and somatosensory discrimination impairments contribute to motor planning deficits and balance difficulties in individuals with DCD (Gauduel et al., 2024; Martel et al., 2022; Smits-Engelsman and Wilson, 2013). Complementing this, reduced tactile sensitivity has been associated with upper limb motor function difficulties in children with DCD (Cox et al., 2015), suggesting that disruptions across the somatosensory spectrum, whether in the direction of heightened or diminished sensitivity, may affect motor coordination. Collectively, these findings suggest that movement difficulties in DCD may reflect imprecise body representation across sensory and motor domains, impairing the internal models required for accurate motor planning (Gauduel et al., 2024; Smits-Engelsman and Wilson, 2013).

Yet, whether SOR persists into midlife, and how it may interact with normative age-related sensory changes, has not been directly examined. Age-related changes in sensory processing have been documented, including altered tactile sensitivity and declining proprioceptive acuity (Decorps et al., 2014; Goble et al., 2009). For individuals with pre-existing SOR, these normative changes may compound existing difficulties, potentially amplifying the sensory-motor challenges characteristic of DCD. Nevertheless, DCD in adulthood remains broadly underrecognized and under-researched (Blank et al., 2019; Verlinden et al., 2023), and virtually no work has examined SOR-DCD symptomatology relationships specifically in midlife adults within community-based samples.

Although converging evidence supports a sensory-motor link (Smits-Engelsman and Wilson, 2013; Tran et al., 2022), this association may not reflect a direct or unconfounded pathway. Keating et al. (2024) found that while children with DCD exhibited sensory differences, motor abilities did not uniquely predict those differences after controlling for ADHD symptoms and autistic traits, both of which independently predicted sensory differences (Keating et al., 2024). This finding underscores the importance of considering co-occurring neurodevelopmental traits when examining sensory-motor relationships. ADHD and autistic traits frequently co-occur with DCD (Blank et al., 2019; Kadesjö and Gillberg, 2003), and each has been independently associated with both sensory processing differences and difficulties in emotion regulation (Graziano and Garcia, 2016; Mazefsky et al., 2013). Given this complexity, the co-occurrence of SOR and DCD symptomatology may be partially attributable to shared neurodevelopmental features rather than to a direct sensory-to-motor mechanism, and alternative mechanisms linking SOR to DCD symptomatology warrant systematic investigation.

One such mechanism may involve difficulties in emotion regulation. This construct must be distinguished from the emotional outcomes with which it has often been conflated in DCD literature: prior research has documented elevated anxiety and depression in DCD populations (Draghi et al., 2020; Harrowell et al., 2017), but these are emotional outcomes rather than regulatory processes. Following Gratz and Roemer (2004), difficulties in emotion regulation encompass: (a) limited awareness and understanding of emotions; (b) non-acceptance of emotional responses; (c) difficulty engaging in goal-directed behavior when distressed; (d) difficulty controlling impulsive behavior when distressed; (e) restricted access to effective regulatory strategies; and (f) lack of emotional clarity (Gratz and Roemer, 2004). These are process-level capacities, distinct from the presence of specific emotional symptoms, and to our knowledge, no prior study has directly examined them in adults with DCD.

The relevance of emotion regulation to DCD specifically is supported by theoretical and empirical considerations. Executive function deficits, which are well-documented in DCD (Blank et al., 2019; Leonard and Hill, 2015), share substantial overlap with the cognitive control processes underlying emotion regulation, particularly inhibition, attention shifting, and goal maintenance (Hofmann et al., 2012; Schmeichel, 2007). When these resources are taxed by emotion regulation demands, fewer remain available for motor planning and execution (Schmeichel, 2007), a trade-off that may be particularly consequential for individuals whose motor control is already compromised. Direct evidence supports this reasoning: Robinson and Klein (2022) demonstrated across three studies that impulse control difficulties, a core dimension of emotion regulation, manifested as measurable motor signatures, specifically as increased tremor instability during fine motor tasks (Robinson and Klein, 2022). Niermeyer et al. (2016) further showed that executive functioning mediated the relationship between habitual expressive suppression and action-planning latencies in young adults, confirming that regulatory demands on executive resources translate into quantifiable motor costs (Niermeyer et al., 2016).

Empirical evidence further supports the relevance of emotion regulation to the SOR-DCD relationship specifically. Difficulties in emotion regulation have been independently associated with SOR (McMahon et al., 2019), and atypical sensory modulation is linked to elevated psychological distress in non-clinical adult populations (Bar-Shalita and Cermak, 2016). These associations appear to reflect shared neurobiological substrates: autonomic nervous system dysregulation and limbic hyperreactivity have been proposed as common mechanisms underlying both SOR and difficulties in emotion regulation (Green and Ben-Sasson, 2010), with direct evidence of amygdala overactivity in individuals with SOR (Green et al., 2015). Prospective research further demonstrates that SOR in childhood predicts subsequent anxiety disorders in a temporally ordered manner (Carpenter et al., 2019), while cross-sectional mediation analyses suggest that difficulties in emotion regulation may represent a key mechanism underlying the SOR-distress association (McMahon et al., 2019). Taken together, this evidence positions difficulties in emotion regulation as a plausible mediating pathway between SOR and DCD symptomatology, one that has not previously been tested.

The present study examines whether difficulties in emotion regulation mediate the association between SOR and DCD symptomatology in community-dwelling midlife adults. We hypothesize that (1) SOR is positively associated with DCD symptomatology, and (2) this association is mediated by difficulties in emotion regulation.

## 2 Methods

### 2.1 Design and participants

This cross-sectional study included a subset of midlife adults from the Jerusalem Perinatal Study (JPS) cohort (Harlap et al., 2007), who participated in the most recent follow-up assessment and consented to this extension study. Data collection occurred between November 2020 and December 2021 (Talisman et al., 2022). Full demographic characteristics of the sample (*N =* 89; 47% women; age range 44.33–46.87 years) are presented in Table 1. All participants were functionally independent, community-dwelling adults: employed, managing daily and family responsibilities.

**Table 1:** Descriptive statistics, reliability coefficients, and correlations with DCD symptomatology (*N =* 89).

Variables	Mean ± SD (range)	Cronbach’s *α*^1^	Correlation with DCD symptomatology (ADC)^2^
Sex	42 women (47%)	–	0.02
Age (years)	45.59 ± 0.69 (44.33–46.87)	–	−0.19
BMI	27.32 ± 4.33 (17.93–41.83)	–	−0.12
SOR	1.94 ± 0.32 (1.23–2.84)	0.70	0.36**
DCD symptomatology	1.71 ± 0.35 (1.23–2.77)	0.89	–
DCD childhood (Subscale A)	1.43 ± 0.43 (1.00–3.00)	–	0.76**
Difficulties in ER	68.49 ± 16.96 (36–124)	0.93	0.58**
Physical activity^3^	Median 135 (0–2,520)	-	−0.23**

BMI, body mass index; SOR, sensory over-responsiveness; DCD, developmental coordination disorder; ER, emotion regulation. Mean scores for SOR and DCD represent untransformed item-level means; rank-based inverse normal transformation was applied to DCD, BMI, and difficulties in ER for regression analyses only. ^1^Cronbach’s α from published validation studies (Bar-Shalita et al., 2009; Gratz and Roemer, 2004; Kirby et al., 2010). ^2^Pearson correlations except where noted; point-biserial for sex. ^3^Spearman’s rho reported due to positive skew. SOR was also significantly correlated with difficulties in ER (*r* = 0.36, *p <* 0.01). **p < 0.01.

Participants received information about the study aims and provided written informed consent before completing an online questionnaire battery. The assessment included three validated instruments: the Adult Developmental Coordination Disorders/Dyspraxia Checklist (ADC; Kirby et al., 2010), the Sensory Responsiveness Questionnaire (SRQ; Bar-Shalita et al., 2009), and the Difficulties in Emotion Regulation Scale (DERS; Gratz and Roemer, 2004). Internal consistency reliability coefficients (Cronbach’s *α*) for each measure are reported in Table 1. Demographic data, anthropometric measurements, and socio-economic status were collected by the JPS study team, encompassing factors such as sex, age, and Body Mass Index (BMI; kg/m^2^). Information on co-occurring neurodevelopmental conditions (e.g., ADHD, autism spectrum disorder) was not systematically collected in the JPS cohort.

Participants were excluded if they: (1) did not complete at least four items on any questionnaire; missing items were prorated using standard procedures (Gratz and Roemer, 2004; Kirby et al., 2010); (2) reported acute illness or musculoskeletal complaints; (3) had chronic conditions affecting physical performance; or (4) were taking medications influencing physical performance. The inclusion criterion was participation in the JPS follow-up at age ~45.

#### 2.1.1 Sample size

The final sample consisted of 89 participants, all available from the JPS cohort who met inclusion criteria. Post-hoc sensitivity analysis (Monte Carlo; a-path r = 0.34, b-path r = 0.58) indicated adequate power (>0.80), consistent with Fritz and MacKinnon (2007) guidelines (Fritz and Mackinnon, 2007).

### 2.2 Measures

#### 2.2.1 Dependent variable: DCD symptomatology

DCD symptomatology was assessed using the Adult Developmental Coordination Disorders/Dyspraxia Checklist (ADC; Kirby et al., 2010), a validated self-report instrument designed for individuals aged 16 years and older that evaluates motor function and coordination in daily contexts, including self-care, fine motor tasks, and occupational functioning.

Following Kirby et al. (2010), the ADC comprises three subscales: subscale A (10 items concerning childhood motor difficulties), subscale B (10 items concerning current self-perception of motor performance), and subscale C (20 items concerning current functional and social consequences of motor difficulties as reflected by others). For the present study, subscales B and C were combined to yield a 30-item measure of current adult motor functioning, while subscale A was excluded because the present study focused on current adult motor symptomatology, consistent with our primary interest in current associations rather than developmental origin. Because childhood onset was not formally established, we use the term “DCD symptomatology” to denote self-reported motor coordination difficulties assessed with a DCD-specific instrument.

Participants rated each item on a 4-point Likert scale according to frequency: “Never” [1], “Sometimes” [2], “Frequently” [3], or “Always” [4], as in the original ADC version (Kirby et al., 2010; Rosenblum, 2013). Scores were computed as item-level means, with the total score divided by the number of items completed, with lower scores indicating better motor performance. Because the present study used subscales B and C as a continuous measure rather than for diagnostic classification, no clinical cut-off values were applied to the data.

#### 2.2.2 Independent variable: sensory over-responsiveness

SOR was assessed using the SRQ-Aversive subscale (Bar-Shalita et al., 2009), a 32-item self-report instrument identifying sensory modulation difficulties through behavioral responses to everyday sensations across six modalities (vestibular, auditory, olfactory, visual, gustatory, somatosensory), excluding pain. Participants rated responses on a 5-point scale (1 = not at all to 5 = very much so, e.g., “eating crunchy foods bothers me”). Scores were computed as item-level means, with higher scores indicating greater SOR. A mean item score of 2.39 (total score ≈ 76.5) has been established as the clinical cut-off distinguishing individuals with clinically significant SOR from the general population (Bar-Shalita et al., 2009). The SRQ has demonstrated strong psychometric properties, including content, construct, and criterion validity; high internal consistency for the SRQ-Aversive subscale (Cronbach’s *α* = 0.90); and good test–retest reliability (r = 0.71, *p <* 0.001; Bar-Shalita et al., 2009; see Table 1).

#### 2.2.3 Mediator variable: difficulties in emotion regulation

Difficulties in emotion regulation were assessed using the DERS (Gratz and Roemer, 2004), a 36-item self-report questionnaire assessing six components: emotional awareness, emotional clarity, impulse control when distressed, goal-directed behavior when distressed, acceptance of emotions, and access to regulation strategies. Participants rated items on a 5-point scale (1 = almost never to 5 = almost always). The total score (range: 36–180) was used, with higher scores indicating greater difficulties in emotion regulation. For reference, Gratz and Roemer (2004) reported M = 77.99 (SD = 20.72) in a non-clinical sample; no clinical cut-off exists as the DERS captures the full continuum of regulatory difficulties (Gratz and Roemer, 2004). The total score was selected because our model conceptualizes difficulties in emotion regulation as a global construct; analyzing subscales separately would inflate Type I error given the sample size. The DERS has demonstrated good test–retest reliability (r = 0.88) and high internal consistency (Gratz and Roemer, 2004; see Table 1).

#### 2.2.4 Covariates

Physical activity was examined as a potential covariate. Participants estimated frequency and duration of moderate and intense physical activities; total weekly minutes were tallied. Physical activity was included given its established associations with both motor functioning and emotion regulation (Lubans et al., 2016; Stillman et al., 2020). Demographic variables (age, sex, BMI) were examined in preliminary correlation analyses; their inclusion in the mediation model was determined based on significant associations with the outcome variable (see Section 3.1).

### 2.3 Data analysis

Data were analyzed using IBM SPSS version 27.0.0.0, with significance set at *p <* 0.05. Descriptive statistics were computed for all variables (SOR, difficulties in emotion regulation, DCD symptomatology, BMI, age, and physical activity) and assessed for normality. Non-normally distributed variables (DCD, BMI, and difficulties in emotion regulation) underwent rank-based inverse normal transformation for regression analyses. Physical activity was positively skewed but not transformed, as it served only as a covariate analyzed using Spearman’s correlation. In OLS-based regression, the normality assumption pertains to the residuals rather than to the predictor variables or covariates (Cohen et al., 2003), and the PROCESS macro further mitigates distributional concerns through bootstrap-based inference (Hayes, 2013).

In the first stage, we conducted bivariate correlation analyses to examine the relationships between: (1) SOR and DCD symptomatology, (2) SOR and difficulties in emotion regulation, and (3) difficulties in emotion regulation and DCD symptomatology. Additionally, we examined correlations between the outcome variable (DCD symptomatology) and demographic/control variables using Pearson’s correlations for continuous variables (age, BMI), a point-biserial correlation for sex (dichotomous variable), and Spearman’s correlation for physical activity due to its positively skewed distribution (see Table 1).

In the second stage, we performed mediation analysis employing Hayes’ PROCESS macro (Version 4.0) for SPSS (Hayes, 2013). Specifically, Model 4 was used to assess the mediating effect of difficulties in emotion regulation on the relationship between SOR and DCD symptomatology. Given the limitations of the sample size for detecting small effects, a bootstrapping method using 5,000 samples was employed; indirect effects were considered significant if the 95% confidence interval did not include zero. Covariates were included in the mediation model only if they showed significant associations with the outcome variable in preliminary analyses.

## 3 Results

The final sample consisted of 89 participants (47% women), aged 45.59 ± 0.69 years (range 44.33–46.87; see Table 1 for full descriptive statistics and demographic characteristics).

### 3.1 Descriptive statistics and correlation analyses

Correlation analyses revealed that DCD symptomatology was significantly positively correlated with both SOR and difficulties in emotion regulation and negatively correlated with physical activity (see Table 1). SOR was also significantly correlated with difficulties in emotion regulation (r = 0.36, *p <* 0.01). Other socio-demographic variables (i.e., sex, age, and BMI) did not show significant associations with DCD symptomatology. Based on these findings, and consistent with the rationale described in Section 2.2.4, only physical activity was included as a covariate in the mediation analysis.

Although the sample size precluded formal mediation testing at the subscale level, exploratory correlational analyses examined the associations between DCD symptomatology and the six DERS subscales. A differential pattern emerged: the subscales most reliant on executive resources showed the strongest associations: Strategies (r = 0.55, *p <* 0.001), Impulse (r = 0.48, *p <* 0.001), and Goals (r = 0.44, *p <* 0.001); whereas the more reflective-cognitive subscales showed weaker, though still significant, associations: Awareness (r = 0.33, *p =* 0.002) and Clarity (r = 0.33, *p =* 0.002). Non-acceptance showed an intermediate association (r = 0.45, *p <* 0.001). All correlations were significant at *p <* 0.01; however, the magnitude gradient, with executive-dependent subscales yielding substantially larger correlations than reflective-cognitive subscales, suggests specificity in which regulatory capacities are associated with motor coordination difficulties.

### 3.2 Mediation analyses

**Figure 1:**
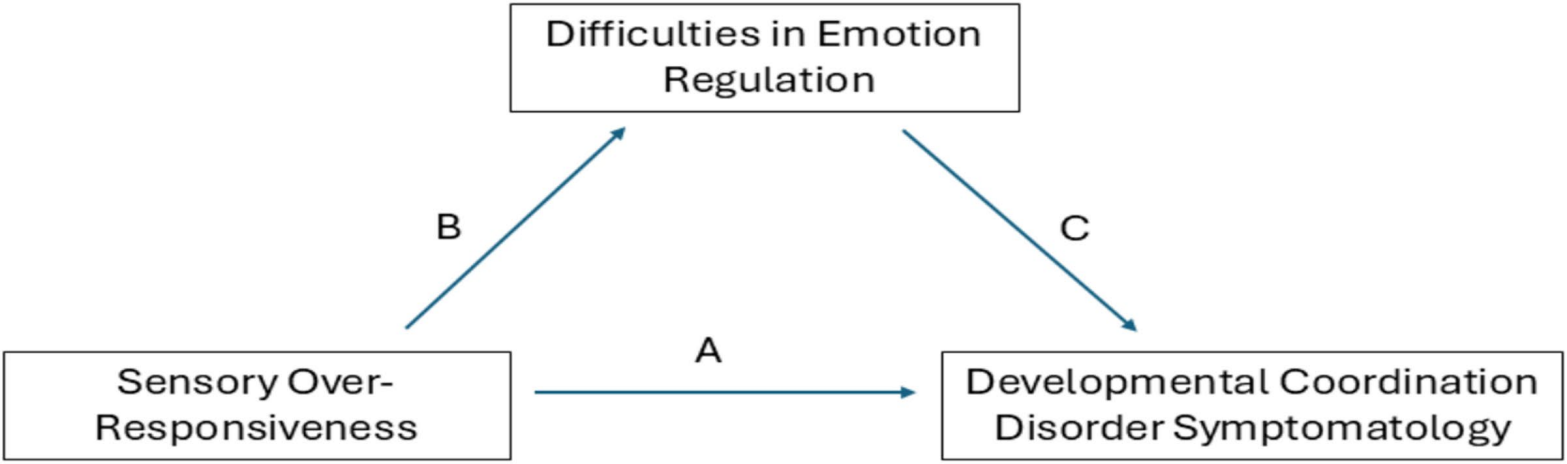
Simple mediation model. Path A = direct effect of SOR on DCD symptomatology controlling for difficulties in emotion regulation (c’); Path B = effect of SOR on difficulties in emotion regulation (a path); Path C = effect of difficulties in emotion regulation on DCD symptomatology controlling for SOR (b path). Physical activity included as covariate.

The proposed mediation model is outlined in Figure 1. Path labels are defined as follows: Path A denotes the direct effect of SOR on DCD symptomatology after controlling for difficulties in emotion regulation (corresponding to path c′ in standard PROCESS notation); Path B denotes the effect of SOR on difficulties in emotion regulation (path a); Path C denotes the effect of difficulties in emotion regulation on DCD symptomatology controlling for SOR (path b). Physical activity was included as a covariate due to its significant negative association with DCD symptomatology (r = −0.23, *p <* 0.01); BMI and age were not included as they showed no significant associations with the outcome variable (see Table 1).

Table 2 presents the full results of the mediation analysis. Mediation modeling indicated that SOR was significantly associated with difficulties in emotion regulation [Path B: B = 18.07, *t* (87) = 3.40, *p =* 0.001]. When controlling for difficulties in emotion regulation, SOR was no longer significantly associated with DCD symptomatology [Path A: B = 0.151, *t* (86) = 1.60, *p =* 0.114]. The total effect of SOR on DCD symptomatology was significant [B = 0.343, *t* (87) = 3.28, *p =* 0.002]. Bootstrap analysis revealed a significant indirect effect of SOR on DCD symptomatology through difficulties in emotion regulation (Path B × Path C: B = 0.192, 95% CI [0.084, 0.323]). Taken together, these results indicate that the association between SOR and DCD symptomatology was fully accounted for by difficulties in emotion regulation. A supplementary mediation analysis incorporating the full ADC score (subscales A, B, and C) yielded substantively identical results, confirming that the conclusions are not sensitive to the inclusion or exclusion of subscale A (see Supplementary material).

**Table 2:** Mediation analysis results.

Path	*B*	*SE*	*t (df)*	*p*	95% CI
Path A: Direct: SOR → DCD	0.151	0.095	1.60 (86)	0.114	[−0.037, 0.339]
Path B: SOR → Difficulties in ER	18.07	5.31	3.40 (87)	0.001	[7.49, 28.65]
Path C: Difficulties in ER → DCD	0.011	0.002	5.50 (86)	< 0.001	[0.007, 0.015]
Indirect effect (B × C)	0.192	0.062	-	-	[0.084, 0.323]
Total effect	0.343	0.105	3.28 (87)	0.002	[0.135, 0.551]

Unstandardized coefficients (B). Bootstrap CIs based on 5,000 resamples. Physical activity included as covariate. ER, emotion regulation. Path A = direct effect of SOR on DCD symptomatology (c′ in PROCESS), controlling for difficulties in ER; Path B = SOR → difficulties in ER (a path); Path C = difficulties in ER → DCD symptomatology (b path), controlling for SOR.

## 4 Discussion

Our findings demonstrate that difficulties in emotion regulation fully mediate the relationship between SOR and DCD symptomatology in a community-based midlife adult sample, after controlling for physical activity. While previous research has identified direct SOR-DCD associations in younger populations (Allen and Casey, 2017; Tran et al., 2022), our results suggest that in midlife, this relationship operates primarily through regulatory pathways rather than direct sensory-motor connections.

The first pathway in this mediation model, from SOR to difficulties in emotion regulation, is consistent with previous research establishing associations between sensory processing differences and regulatory difficulties across the lifespan (McMahon et al., 2019), as well as links to broader emotional outcomes such as anxiety (Ben-Sasson et al., 2009). Two converging mechanisms have been proposed to explain this link. First, individuals who experience heightened sensory responsiveness must continually manage their responses to environmental stimuli, placing chronic demands on regulatory systems (Bar-Shalita and Cermak, 2016). Second, atypical sensory processing has been empirically linked to maladaptive regulatory patterns: sensory sensitivity has been associated with an emotion-focused coping style (Jerome and Liss, 2005). Adults with sensory over-responsiveness further exhibit elevated anxiety and depression and reduced psychosocial functioning (Kinnealey et al., 2011). Similar avoidance-based coping has been documented in adults with DCD (Meachon and Alpers, 2023), suggesting convergent regulatory challenges across sensory and motor difficulties that may increase cumulative regulatory burden over time.

Our findings are consistent with these proposed mechanisms, though the cross-sectional design precludes establishing which pathway is operative.

The second pathway, from difficulties in emotion regulation to DCD symptomatology, can be understood within a resource-competition framework. Schmeichel (2007) demonstrated that committing cognitive resources to emotion regulation diminishes executive functioning in healthy adults (Schmeichel, 2007). Although no prior study has directly examined this association in adults with DCD symptomatology, our significant Path C is consistent with this framework. Additional evidence provides context: Biotteau et al. (2019) demonstrated that individuals with DCD exhibit slower processing and reduced motor automatization, making motor performance more cognitively demanding (Biotteau et al., 2019), while Coombes et al. (2009) showed that emotional arousal impaired motor efficiency specifically at force levels requiring greater attentional control (Coombes et al., 2009). Taken together, these studies suggest a plausible mechanism by which difficulties in emotion regulation could interfere with motor coordination. However, our findings do not imply that difficulties in emotion regulation cause DCD; rather, they may compound existing motor vulnerabilities by reducing available cognitive resources.

Further support for this resource-competition account emerges from subscale-level analyses of the DERS, which assesses distinct dimensions of regulatory difficulties (Gratz and Roemer, 2004). Subscales measuring limited access to effective regulatory strategies (Strategies), difficulties controlling impulsive behaviors when experiencing negative emotions (Impulse), and difficulties engaging in goal-directed behavior when upset (Goals) showed stronger associations with DCD symptomatology than subscales measuring lack of emotional awareness (Awareness) and lack of emotional clarity (Clarity). This differential pattern aligns with emerging evidence that specific DERS subscales relate to distinct executive processes: Furente et al. (2024) found that cognitive proficiency discrepancies predicted Goals, Strategies, and Clarity subscales in adolescents with internalizing disorders (Furente et al., 2024). Malagoli and Usai (2018) demonstrated that working memory efficiency was negatively associated with difficulties in accessing regulatory strategies (Malagoli and Usai 2018). Marceau et al. (2018) further demonstrated that among the three basic executive functions, task-switching, the process most closely linked to cognitive flexibility, was uniquely associated with difficulties in emotion regulation, while working memory and inhibition were not, suggesting differential involvement of executive processes in regulatory capacity (Marceau et al., 2018). Taken together, these findings suggest that different DERS subscales relate to distinct executive processes (Furente et al., 2024; Malagoli and Usai, 2018; Marceau et al., 2018). Our finding that subscales linked to effortful control showed preferential associations with DCD symptomatology, whereas subscales assessing basic awareness capacities did not, argues against the interpretation that the mediation effect reflects general emotional distress. However, executive functions were not measured directly in the present study, and future research incorporating objective measures is needed to test this account.

These findings can be situated within the broader theoretical literature on DCD and emotional functioning. The Environmental Stress Hypothesis (ESH; Cairney et al., 2013) proposes that motor difficulties generate chronic stress that erodes emotional functioning, a framework that has received substantial empirical support (Omer et al., 2019) and has recently been extended to adult populations (Erskine et al., 2024; Mancini et al., 2016). Our findings offer a complementary perspective by identifying a pathway in the opposite direction: SOR contributing to difficulties in emotion regulation, which in turn relate to DCD symptomatology. These constructs differ in important ways: whereas the ESH focuses on chronic stress as an outcome of motor difficulties, our model examines difficulties in emotion regulation, the capacity to modulate emotional responses, as a mediating process. These are related but distinct constructs (Gross, 2015). The ESH itself predicts bidirectionality between motor and emotional functioning, and Miller et al. (2023) provided experimental support for such bidirectionality in a randomized controlled trial demonstrating that a motor competence intervention improved behavioral self-regulation (Miller et al., 2023). Together with our findings, this suggests reciprocal influences between motor and regulatory domains that warrant longitudinal investigation.

The present findings emerged in a midlife sample. As a developmental context, midlife involves neurobiological changes including reductions in prefrontal-subcortical white matter integrity (Salthouse, 2009; Seidler et al., 2010), hormonal transitions such as perimenopausal estrogen fluctuation and gradual testosterone decline (Weber et al., 2014; Yeap, 2009), and competing psychosocial demands (Infurna et al., 2020; Lachman et al., 2015). However, whether such factors are specifically relevant to the mediation pattern observed here cannot be determined from the present data and requires empirical testing across age groups.

These findings have potential clinical implications. Assessments of DCD symptomatology in midlife adults may benefit from routine evaluation of sensory processing and emotion regulation, consistent with growing recognition that DCD assessment should account for the broader functional profile across the lifespan (Blank et al., 2019; Tal Saban and Kirby, 2018). The mediation pattern also raises the possibility that interventions targeting emotion regulation, such as mindfulness-based stress reduction (Kabat-Zinn, 2003), cognitive-behavioral strategies (Berking and Wupperman, 2012), or acceptance and commitment therapy (Hayes et al., 2006), could support midlife adults experiencing DCD symptomatology in the context of SOR. Moreover, given the frequent co-occurrence of DCD with ADHD and autistic traits (Kadesjö and Gillberg, 2003; Keating et al., 2024) and their independent links to both sensory and regulatory difficulties (Graziano and Garcia, 2016; Mazefsky et al., 2013), clinical assessments in midlife adults should also screen for these neurodevelopmental features, thereby informing intervention planning. However, these implications should be interpreted cautiously: the cross-sectional design cannot establish whether targeting emotion regulation would improve motor outcomes, and current DCD guidelines emphasize supporting functionality rather than symptom reduction (Blank et al., 2019).

Several limitations warrant consideration. First, the cross-sectional design limits causal inference; alternative causal orderings, including motor difficulties contributing to difficulties in emotion regulation, consistent with the ESH, remain plausible. Our reliance on self-report measures raises concerns about shared method variance, as both the DERS and ADC are self-report instruments. Future research should incorporate objective motor assessments, though self-report remains appropriate for subjective sensory and emotional experiences. The interpretation of subscale-level findings draws on literature linking specific DERS subscales to executive processes, but executive functions were not measured directly; this association requires confirmation with objective cognitive measures. Although exploratory subscale-level correlations revealed a differential pattern, the DERS total score was used for the primary mediation analysis because the sample size precluded reliable subscale-level mediation testing. Similarly, the neurobiological and psychosocial account of the complete mediation pattern was not directly tested and remains to be validated empirically.

Sample characteristics impose further constraints. The relatively narrow age range restricts the generalizability of the findings to other adult age groups, and the absence of data on co-occurring neurodevelopmental diagnoses such as ADHD or autistic characteristics, which have been linked in their own right to both sensory processing differences and difficulties in emotion regulation (Graziano and Garcia, 2016; Keating et al., 2024; Mazefsky et al., 2013), prevents isolating a sensory-specific pathway from broader neurodevelopmental contributions. Subsequent research should systematically assess ADHD and autistic traits using validated screening instruments to clarify their role, whether as confounds, moderators, or additional mediators, in the association between SOR, emotion regulation, and motor coordination difficulties.

Notwithstanding these limitations, the present study advances current understanding of sensory-regulatory-motor associations in several interrelated respects. Drawing on a community-based birth cohort of midlife adults, rather than a clinically defined sample, the findings demonstrate that the link between sensory over-responsiveness and DCD symptomatology operates within the typical, non-pathological range of the general population, supporting dimensional conceptualizations of neurodevelopmental phenotypes (Astle et al., 2022). Within this framework, the identification of difficulties in emotion regulation as a complete mediator, conceptually distinct from emotional outcomes such as anxiety and depression with which it has often been conflated in the DCD literature (Draghi et al., 2020), refines theoretical accounts of how sensory and motor difficulties cohere across adulthood. The differential pattern observed across DERS subscales, with executive-dependent dimensions showing the strongest associations with DCD symptomatology, further specifies the mechanistic account by implicating effortful control rather than general emotional distress. Together, these contributions establish midlife adulthood, examined here for the first time within a general-population cohort, as a developmental window in which regulatory processes appear to organize the sensory-motor relationship in ways not yet captured by existing models (Erskine et al., 2024; Tal Saban and Kirby, 2018).

In conclusion, the present study demonstrates that difficulties in emotion regulation fully mediate the association between sensory over-responsiveness and DCD symptomatology among midlife adults, with this pattern remaining robust after controlling for physical activity. The findings extend the Environmental Stress Hypothesis, which has emphasized motor difficulties as a source of cumulative emotional strain, by identifying an additional route in which sensory and regulatory processes contribute to motor outcomes. Observed in a community-based sample, this pattern positions midlife as a critical period for understanding how regulatory processes shape motor coordination across adulthood, including in adults whose difficulties may not reach formal diagnostic thresholds. Future research should employ longitudinal designs to test the directionality of these regulatory pathways and evaluate whether interventions targeting emotion regulation can complement traditional motor-focused approaches across the full spectrum of motor coordination difficulties in adulthood.

## Data Availability

The raw data supporting the conclusions of this article will be made available by the authors, without undue reservation.
