# Supplementary material for: Difficulties in emotion regulation mediate the association between sensory over-responsiveness and motor coordination symptoms in community-dwelling midlife adults: a cross-sectional analysis
Source: Front Psychol. 2026 May 29;17:1779854. doi: 10.3389/fpsyg.2026.1779854 (PMC13260136; doi:10.3389/fpsyg.2026.1779854)
Supplement: Supplementary file 1 [file Supplementary_file_1.PDF]

Supplementary Materials

Difficulties in emotion regulation mediate the association between sensory over-responsiveness and motor coordination symptoms in community-dwelling midlife adults: A cross-sectional analysis

S1. Robustness check: Mediation analysis using the full ADC score

In the primary analysis reported in the manuscript, DCD symptomatology was operationalized as the mean of subscales B and C of the Adult Developmental Coordination Disorders/Dyspraxia Checklist (ADC; Kirby et al., 2010), focusing on current adult motor symptomatology. To examine whether the substantive findings depended on this operationalization, we conducted a robustness check using the full ADC score, which additionally incorporates subscale A (10 items concerning childhood motor difficulties).

The mediation analysis was re-run using PROCESS Model 4 (Hayes, 2013) with 5,000 bootstrap resamples and physical activity included as a covariate, substituting the full ADC score (subscales A + B + C) for the subscale B+C composite as the outcome variable.

Results

The pattern of results was substantively unchanged. Sensory over-responsiveness predicted difficulties in emotion regulation, which in turn predicted DCD symptomatology measured by the full ADC. The total effect of SOR on DCD symptomatology remained statistically significant, the indirect effect through difficulties in emotion regulation remained significant with a 95% bootstrap confidence interval that excluded zero, and the direct effect remained non-significant, consistent with full mediation. Detailed coefficients are presented in Table S1.

Table S1. *Comparison of mediation results between the primary analysis (subscales B+C) and the robustness check (full ADC).*

**Path / Effect**	**Primary analysis (subscales B+C)**	**Robustness check (full ADC)**
Path a (SOR → DERS)	B = 18.07, p = .001	B = 16.63, p = .002
Path b (DERS → DCD)	B = 0.011, p < .001	B = 0.008, p < .001
Total effect c (SOR → DCD)	B = 0.343, p = .002	B = 0.272, p = .006
Direct effect c′ (SOR → DCD | DERS)	B = 0.151, p = .114	B = 0.136, p = .147
Indirect effect a×b	**B = 0.192**	**B = 0.136**
95% bootstrap CI for indirect effect	[0.084, 0.323]	[0.050, 0.256]

Note. SOR = sensory over-responsiveness; DERS = Difficulties in Emotion Regulation Scale total score; DCD = Developmental Coordination Disorder symptomatology. Coefficients are unstandardized and were computed using PROCESS Model 4 (Hayes, 2013) in SPSS, with 5,000 bootstrap resamples for the

indirect effect. The primary analysis (subscales B+C) is reported in the main manuscript. The robustness check (full ADC, subscales A+B+C) was conducted on the same sample and included physical activity as a covariate.

Conclusion

The robustness check confirms that the mediation pattern reported in the manuscript holds when DCD symptomatology is operationalized to include retrospective childhood motor difficulties (subscale A) alongside current adult motor symptomatology. The direction and significance of all effects, including the full mediation pattern, were unchanged. The decision to operationalize DCD symptomatology as the mean of subscales B and C in the primary analysis, focusing on current adult motor symptomatology, did not affect the substantive conclusions of the study.

References

Hayes, A. F. (2013). *Introduction to mediation, moderation, and conditional process analysis: A regression-based approach*. Guilford Press.

Kirby, A., Edwards, L., Sugden, D., & Rosenblum, S. (2010). The development and standardization of the Adult Developmental Co-ordination Disorders/Dyspraxia Checklist (ADC). *Research in Developmental Disabilities, 31*(1), 131-139. https://doi.org/10.1016/j.ridd.2009.08.010
